# Characterisation of tree nut allergy in a population in a centre in the North of Portugal

**DOI:** 10.1186/2045-7022-5-S3-P66

**Published:** 2015-03-30

**Authors:** Fabrícia Carolino, Diana Silva, Mariana Couto, Alice Coimbra, José Luís Plácido

**Affiliations:** 1 Serviço de Imunoalergologia, Centro Hospitalar São João, Porto, Portugal; 2 Laboratório de Imunologia, Faculdade de Medicina, Universidade do Porto, Porto, Portugal

## Background

Tree nuts and peanuts are among the major causes of food allergy (FA) in both children and adults. Cross-reactivity is common among different nuts, but may also occur with peanuts, although they are unrelated foods. We aim to describe the prevalence and characteristics of tree nut allergy among children and adults followed in a University Hospital Allergy Department in the North of Portugal.

## Methods

Medical records of patients studied in our Food Allergy Unit between Jan/2011 and Dec/2013 were reviewed; 134 children (55% male; median age 7y [IQR 3-13y]) and 198 adults (24% male; median age 36y [IQR 27-46y]) were studied for 194 and 315 suspected food allergic reactions, respectively. For those with suspected tree nut and/or peanut allergy, we recorded demographic characteristics, clinical history and study results based on sIgE, skin tests (ST), and oral food challenges (OFC).

## Results

Fifty-two patients, 12 (9%) children and 40 (20%) adults, were referred for 65 suspected tree nut and/or peanut allergies (13% of all reactions evaluated). FA to tree nuts was confirmed in 26 cases (73% adults), representing 8% of all patients studied, based on clinical history and positive ST and/or sIgE (n=23) or positive OFC (n=3). The main presentations were anaphylaxis in 13 cases (67% of the children vs. 47% adults, p=0.645) and isolated mucocutaneous involvement in 12 cases (33% vs. 53%, p=0.645); 60% were immediate reactions. The main culprit was walnut in 11 cases and, in another 12, more than 1 tree nut was involved. The prevalence of atopy was 87% (83% children vs. 88% adults) and of asthma 42%. All 6 confirmed cases of peanut allergy were sensitized to at least one tree nut but, inversely, only 6 of the 26 (23%) tree nut allergic were also sensitized to peanut.

## Conclusion

The prevalence of tree nut allergy in the population studied in our Food Allergy Unit was of 8%, predominantly affecting adults (73%) and older children. No significant age-related differences were found. In this series, all peanut allergic individuals were also sensitized to nuts, but not the inverse. The majority of the reactions reported were severe.

